# Finerenone-Empagliflozin Combination Therapy in Apparent Resistant Hypertension: A CONFIDENCE Trial Post Hoc Analysis

**DOI:** 10.1016/j.jacadv.2026.103388

**Published:** 2026-10-23

**Authors:** Rajiv Agarwal, Sankar D. Navaneethan, Ricardo Correa-Rotter, Michelle M. Estrella, Hiddo J.L. Heerspink, Janet B. McGill, Amy K. Mottl, Masaomi Nangaku, Julio Rosenstock, Peter Rossing, Muthiah Vaduganathan, Javier Morales, Yehuda Handelsman, Jennifer B. Green, Johannes F.E. Mann, Charlie Scott, Li Li, Meike Brinker, Matthew R. Weir

**Affiliations:** a Division of Nephrology, Richard L. Roudebush VA Medical Center & Indiana University School of Medicine, Indianapolis, Indiana, USA; b Section of Nephrology, Baylor College of Medicine, Houston, Texas, USA; c Department of Nephrology and Mineral Metabolism, National Medical Science and Nutrition Institute Salvador Zubirán, Mexico City, Mexico; d Division of Nephrology and Kidney Health Research Collaborative, University of California, San Francisco, California, USA; e Department of Clinical Pharmacy and Pharmacology, University Medical Center Groningen, University of Groningen, Groningen, the Netherlands; f Division of Endocrinology, Metabolism & Lipid Research, Washington University in St. Louis, School of Medicine, St. Louis, Missouri, USA; g University of North Carolina Kidney Center, UNC School of Medicine, Chapel Hill, North Carolina, USA; h Division of Nephrology and Endocrinology, The University of Tokyo Graduate School of Medicine, Tokyo, Japan; i Department of Medicine, University of Texas Southwestern Medical Center, Dallas, Texas, USA; j Steno Diabetes Center Copenhagen, Copenhagen, and Department of Clinical Medicine, University of Copenhagen, Copenhagen, Denmark; k Division of Cardiovascular Medicine, Brigham and Women's Hospital, and Harvard Medical School, Boston, Massachusetts, USA; l Donald and Barbara Zucker School of Medicine, Hofstra/Northwell University, Hempstead, New York, USA; m Metabolic Institute of America, Tarzana, California, USA; n Division of Endocrinology, Department of Medicine and Duke Clinical Research Institute, Duke University School of Medicine, Durham, North Carolina, USA; o KfH Kidney Centre, Munich, and Department of Nephrology & Hypertension, Friedrich Alexander University, Erlangen, Germany; p Clinical Statistics and Analytics, Bayer US LLC, Whippany, New Jersey, USA; q Global Medical Evidence, Bayer AG, Berlin, Germany; r Cardiology and Nephrology Clinical Development, Bayer AG, Germany; s Division of Nephrology, Department of Medicine, University of Maryland School of Medicine, Baltimore, Maryland, USA

**Keywords:** apparent resistant hypertension, chronic kidney disease, hyperkalemia, nonsteroidal MRA, SGLT2 inhibitor, type 2 diabetes

## Abstract

**Background:**

Apparent treatment-resistant hypertension (aTRH) is common in chronic kidney disease (CKD) with type 2 diabetes and predicts worse outcomes. Spironolactone, the established fourth-line agent, is limited by hyperkalemia in CKD.

**Objectives:**

This analysis assessed whether finerenone plus empagliflozin lowers blood pressure (BP) meaningfully in aTRH, and whether aTRH modifies these agents' efficacy and safety.

**Methods:**

Post hoc analysis of CONFIDENCE (NCT05254002), a double-blind randomized trial of finerenone, empagliflozin, or both in CKD with albuminuria and type 2 diabetes. aTRH was BP ≥130/80 mm Hg on 3 antihypertensive drugs, or ≥4 drugs regardless of BP. Mixed models and logistic regression identified predictors of BP response.

**Results:**

Of the 796 participants, 307 (39%) had aTRH; they were older, more obese, and had more atherosclerotic cardiovascular disease, higher albuminuria, and higher systolic BP (140.9 vs 131.5 mm Hg) but similar estimated glomerular filtration rate (53 vs 55 mL/min/1.73 m^2^). Combination therapy lowered systolic BP more than either monotherapy. Independent predictors of a ≥10 mm Hg systolic BP fall were aTRH (OR: 1.56; 95% CI: 1.08-2.25) and higher baseline estimated glomerular filtration rate (OR: 1.15 per 10 mL/min/1.73 m^2^; 95% CI: 1.05-1.26); diuretic use and albuminuria were not. The aTRH effect was treatment-independent (interaction *P* = 0.21). Adverse events were more frequent with aTRH (63.8% vs 51.1%), yet on combination therapy hyperkalemia was less frequent (11.2% vs 17.5%).

**Conclusions:**

aTRH affected approximately 40% of participants and marked a higher-risk phenotype responsive to finerenone-empagliflozin, with BP reductions comparable to spironolactone but without the excess hyperkalemia that limits its use in CKD.

Apparent treatment-resistant hypertension (aTRH) is a high-risk clinical phenotype defined as blood pressure (BP) that remains above target despite the concurrent use of 3 antihypertensive drug classes, or BP that requires ≥4 medications to achieve control.^1^, ^2^ Although true resistant hypertension requires confirmation by 24-hour ambulatory BP monitoring,^1^, ^3^, ^4^ and objective assessment of medication adherence,^1^, ^5^, ^6^, ^7^ these standards are seldom applied in large-scale cardiovascular trials.^8^, ^9^, ^10^, ^11^, ^12^ Consequently, aTRH (defined by medication count and office BP) remains the operational standard in clinical practice and in the present analysis.

The global burden of hypertension in people aged 30 to 79 years doubled between 1990 and 2019, to affect 1.28 billion adults worldwide in 2019.^13^ In the treated hypertensive population, the prevalence of aTRH, for example, is reported in a review as approximately 14.7%, rising to 28.8% among people with chronic kidney disease (CKD).^14^ In a 2022 report from 2 large U.S. health care systems, 35% to 55% of people with CKD demonstrated aTRH depending on the definition used.^15^ Beyond the challenge of controlling BP, aTRH independently predicts cardiovascular disease and kidney disease events.^16^, ^17^

TRH in CKD is also driven by impaired kidney sodium excretion and volume expansion, which are promoted by several factors.^18^ Two of these factors are of particular importance. First is the activation of the mineralocorticoid receptor (MR) through multiple mechanisms such as aldosterone excess, cortisol excess, and small GTPase Rac1.^19^, ^20^ Second is T-cell–mediated inflammation that promotes inappropriate sodium reabsorption in the distal nephron.^21^, ^22^ The central role of MR overactivation in TRH^23^ explains why the PATHWAY-2 trial established spironolactone as the most effective fourth-line agent for aTRH.^8^ However, spironolactone use in CKD is substantially limited by hyperkalemia, and even with the potassium binder patiromer, the AMBER trial required complex co-administration to enable its use.^9^ A mechanistically complementary approach to overcoming treatment resistance may be possible with the combination of finerenone and empagliflozin. Finerenone, a nonsteroidal MR antagonist (MRA), has the ability to block inflammation^19^ and elicits less hyperkalemia than spironolactone in people with CKD and TRH.^24^ Preclinical studies have shown that finerenone also blocks profibrotic signaling,^25^, ^26^ which drives albuminuria progression in CKD.^27^ Empagliflozin, on the other hand, lowers BP through natriuresis and sympathetic suppression.^28^, ^29^ Furthermore, empagliflozin reduces hyperkalemia risk with steroidal MRAs in heart failure^30^, ^31^; however, when "potassium stress" is small, such as with finerenone, mitigating this risk with the combination may not be detectable.^32^

The CONFIDENCE (Combination Effect of Finerenone and Empagliflozin in Participants with Chronic Kidney Disease and Type 2 Diabetes Using a Urinary Albumin-to-Creatinine Ratio Endpoint) trial—a double-blind, randomized, active-controlled trial of participants with CKD, albuminuria, and type 2 diabetes (T2D)—demonstrated that combination finerenone plus empagliflozin reduced the urinary albumin-to-creatinine ratio (UACR) approximately 30% more than either agent alone.^33^ In people with T2D and CKD, both agents are established cornerstones of guideline-directed cardiorenal therapy, based on their proven reduction of cardiovascular and kidney events.^34^ A complementary analysis from CONFIDENCE demonstrated that baseline systolic BP is the strongest predictor of the magnitude of BP response to these agents.^35^ However, whether this combination delivers clinically meaningful antihypertensive benefit specifically in people who present with the functional phenotype of aTRH (for whom clinicians lack safe therapeutic alternatives to spironolactone in CKD) and whether aTRH status modifies the safety profile of these agents have not been evaluated. In this post hoc analysis of the CONFIDENCE trial, we characterize the clinical phenotype of aTRH in this high-risk population; identify independent predictors of BP response to finerenone, empagliflozin, and their combination and assess whether aTRH modifies the antihypertensive efficacy or safety of these therapies.

## Methods

### Study population and design

The study population consisted of participants from the CONFIDENCE trial (NCT05254002).^33^ This was a double-blind, randomized, active-controlled trial evaluating finerenone alone, empagliflozin alone, or the combination of the 2 therapies in participants with CKD, albuminuria, and T2D. The trial enrolled participants with estimated glomerular filtration rate (eGFR) 30 to 90 mL/min/1.73 m^2^. Participants were excluded if their BP was below 90 mm Hg systolic or above 160/100 mm Hg on day 1. The use of an angiotensin-converting enzyme inhibitor or angiotensin-receptor blocker at maximally tolerated dose was an inclusion criterion.

The trial was conducted in accordance with the Declaration of Helsinki and the International Council for Harmonisation Good Clinical Practice guidelines. There was no single central review body; the protocol and the informed consent documents were reviewed and approved by the institutional review board or independent ethics committee at each participating site. All participants provided written informed consent before any trial procedure.

### Definition of aTRH

For the purposes of this study, we defined aTRH as baseline BP ≥ 130 mm Hg systolic despite taking adequate doses of 3 antihypertensive drug classes or any BP that required ≥4 drugs for treatment.

### Blood pressure measurement

BP in the CONFIDENCE trial was consistently measured in a seated position after at least 5 minutes of rest and was obtained 3 times at every trial visit. The mean of the 3 measurements was documented.

### Outcomes

The outcomes of the present analysis were the change in systolic BP following randomization (days 14, 30, 90, 180, and 210) and the percentage of participants with a ≥10 mm Hg drop in systolic BP at day 180.

### Statistical analysis

The analysis comprised 3 components. First, baseline characteristics were described using descriptive statistics, separately for participants with and without aTRH. Second, predictors of BP response were assessed in the full cohort (N = 796) using 2 complementary approaches: logistic regression modeled the odds of achieving a ≥10 mm Hg reduction in systolic BP at day 180 (719 participants with a day 180 measurement and complete covariates) and linear mixed models estimated the mean trajectory of systolic BP change from baseline through day 210 (767 participants contributing 3,646 observations). Third, to test whether aTRH modifies the efficacy of combination therapy (heterogeneity of treatment effect), a treatment × aTRH interaction term was included in the mixed model.

Baseline characteristics were compared between participants with and without aTRH using absolute standardized differences rather than significance tests, because current guidance discourages hypothesis testing of baseline characteristics; a standardized difference above 0.10 was taken to indicate a meaningful imbalance. For UACR, the standardized difference was computed on the natural log scale, and for variables with more than 2 categories the multivariate standardized difference was used. Adverse event frequencies were compared using the chi-square test, or the Fisher exact test where fewer than 5 events occurred in either group; these tests were 2-sided.

The linear mixed models included a random intercept for patient and fixed-effect indicator variables for each follow-up visit (days 14, 30, 90, 180, and 210). For both the linear mixed models and logistic regression, the same covariates were used: baseline eGFR, UACR, diuretic use, randomized treatment, and the presence or absence of aTRH. Baseline UACR was ln-transformed for all analyses. Baseline systolic BP was deliberately not included as a covariate, because aTRH is operationally defined by elevated baseline systolic BP despite ≥3 antihypertensive agents; conditioning on baseline systolic BP would partial out a constituent of the exposure definition and attenuate the aTRH coefficient, analogous to the overadjustment bias described by Schisterman et al.^36^

To test the robustness of the primary findings, 3 sensitivity analyses were performed. First, age, sex, race, body mass index (BMI), and prior atherosclerotic cardiovascular disease were added as covariates to the mixed model and to the logistic regression model. Because 4 participants in sparsely represented race categories perfectly predicted nonresponse, the logistic model was additionally fitted with geographic region in place of race, and the base model was refitted on each corresponding subset so that adjustment could be distinguished from the change in sample (Supplemental Table 3). Second, the aTRH definition was revised to require a diuretic among the 3 antihypertensive medications (the conventional guideline definition of resistant hypertension), and the mixed model was re-estimated.

Treatment-emergent adverse events were reported for participants who had received at least 1 dose of a trial drug and had an adverse event that had started or worsened after the first dose and up to 3 days after any temporary or permanent interruption of the trial treatment. Adverse events were summarized by treatment group and by aTRH subgroup.

## Results

### Characteristics of participants with aTRH

Table 1 shows the clinical characteristics associated with aTRH vs no aTRH. Participants with aTRH tended to be older; were less often from Asia; and had a higher BMI, a higher prevalence of atherosclerotic cardiovascular disease, and higher UACR compared with those without aTRH. By definition, baseline systolic BP was much higher in participants with than without aTRH, as was antihypertensive drug use. All classes of antihypertensive drugs except angiotensin-converting enzyme inhibitor or angiotensin-receptor blockers were more frequently used in people with aTRH compared with those without aTRH. Of the 307 participants classified as having aTRH, the overwhelming majority (272, 89%) met the criterion of systolic BP ≥ 130 mm Hg on ≥3 antihypertensive medications, while only 35 (11%) were classified due to receiving ≥4 medications with controlled BP.

**Table 1:** Baseline Characteristics of CONFIDENCE Participants by Resistant Hypertension Subgroup

	No aTRH (n = 489, 61%)	aTRH (n = 307, 39%)	Standardized Difference
Systolic BP ≥130 mm Hg, n (%)	256 (52)	272 (89)	—
Systolic BP 90-<130 mm Hg, n (%)	233 (48)	35 (11)	
Treatment group, n (%)			0.065
Combination	160 (33)	107 (35)	
Finerenone	160 (33)	103 (34)	
Empagliflozin	169 (35)	97 (32)	
Age(y), mean (SD)	65.2 (10.4)	68.5 (9.8)	0.330
Male, n (%)	374 (76)	224 (73)	0.081
Geographic region, n (%)			0.380
North America	127 (26)	97 (32)	
Europe	107 (22)	105 (34)	
Asia	255 (52)	105 (34)	
Body mass index (kg/m^2^), mean (SD)	28.3 (5.8)	30.9 (6.2)	0.432
Serum potassium value (mmol/L), mean (SD)	4.5 (0.4)	4.4 (0.4)	0.219
Systolic BP (mm Hg), mean (SD)	131.5 (13.2)	140.9 (11.3)	—
History of atherosclerotic cardiovascular disease, n (%)	101 (21)	122 (40)	0.425
eGFR (mL/min/1.73 m^2^), mean (SD)	55 (17)	53 (17)	0.127
KDIGO CKD stage, n (%)			0.200
G1 or G2	184 (38)	92 (30)	
G3A	140 (29)	90 (30)	
G3B	145 (30)	114 (38)	
G4	19 (4)	8 (3)	
UACR (mg/g), median (25th, 75th percentile)	557 (267, 1,114)	636 (337, 1,096)	0.135
Severity of albuminuria, n (%)			0.166
<300 mg/g	138 (28)	65 (21)	
300-<1,000 mg/g	220 (45)	155 (50)	
1,000-<5,000 mg/g	131 (27)	87 (28)	
HbA_1C_ (%), mean (SD)	7.3 (1.2)	7.3 (1.2)	0.018
Concomitant medications, n (%)			
Statins	350 (72)	247 (80)	0.209
Insulin	184 (38)	133 (43)	0.116
GLP-1 RAs	93 (19)	89 (29)	0.235
Antihypertensive drugs	484 (99)	307 (100)	—
Number of antihypertensive drugs, mean (SD)	1.8 (0.7)	3.6 (0.7)	—
Antihypertensive drugs used, n (%)			
ACEI or ARB	482 (99)	305 (99)	0.077
Beta blockers	68 (14)	213 (69)	1.361
Centrally acting agents	4 (1)	33 (11)	0.435
Calcium-channel blockers	229 (47)	263 (86)	0.901
Alpha blockers	20 (4)	96 (31)	0.762
Diuretic agents	86 (18)	203 (66)	1.130

ACEI = angiotensin-converting enzyme inhibitor; ARB = angiotensin-receptor blocker; aTRH = apparent treatment-resistant hypertension; BP = blood pressure; CKD = chronic kidney disease; eGFR = estimated glomerular filtration rate; G = glomerular filtration rate stage; GLP-1 RA = glucagon-like peptide-1 receptor agonist; HbA1C = glycated hemoglobin; KDIGO = Kidney Disease: Improving Global Outcomes; pc = percentile; UACR = urinary albumin-to-creatinine ratio.

All values are computed in the 796 participants included in this analysis. The final column is the absolute standardized difference between participants with and without aTRH; a value above 0.10 is conventionally taken to indicate a meaningful imbalance. For UACR it is computed on the natural log scale, and for variables with more than 2 categories it is the multivariate standardized difference. Standardized differences are reported in place of significance tests because current guidance discourages hypothesis testing of baseline characteristics. Rows marked—are components of the aTRH definition, which is systolic BP ≥130 mm Hg on 3 antihypertensive drugs or ≥4 drugs regardless of BP, so a between-group contrast there restates the definition; the individual antihypertensive drug class rows are likewise driven by that drug count requirement.

### BP response to treatment

The percent of participants achieving ≥10 mm Hg systolic BP reduction at day 180 (responders) is shown in Figure 1; overall, a greater percentage of participants were BP responders with combination therapy compared with either monotherapy, and the percentage of responders was greater in the aTRH group compared with the no aTRH group. In the logistic regression model of this outcome (Figure 2), combination therapy, aTRH (OR: 1.56; 95% CI: 1.08-2.25; *P* = 0.019), and higher baseline eGFR (OR: 1.15 per 10 mL/min/1.73 m^2^; 95% CI: 1.05-1.26; *P* = 0.003) were independently associated with a systolic BP response of at least −10 mm Hg, whereas diuretic use (OR: 1.09; 95% CI: 0.75-1.58; *P* = 0.70) and baseline UACR (OR: 0.95; 95% CI: 0.80-1.12; *P* = 0.50) were not.

**Figure 1:**
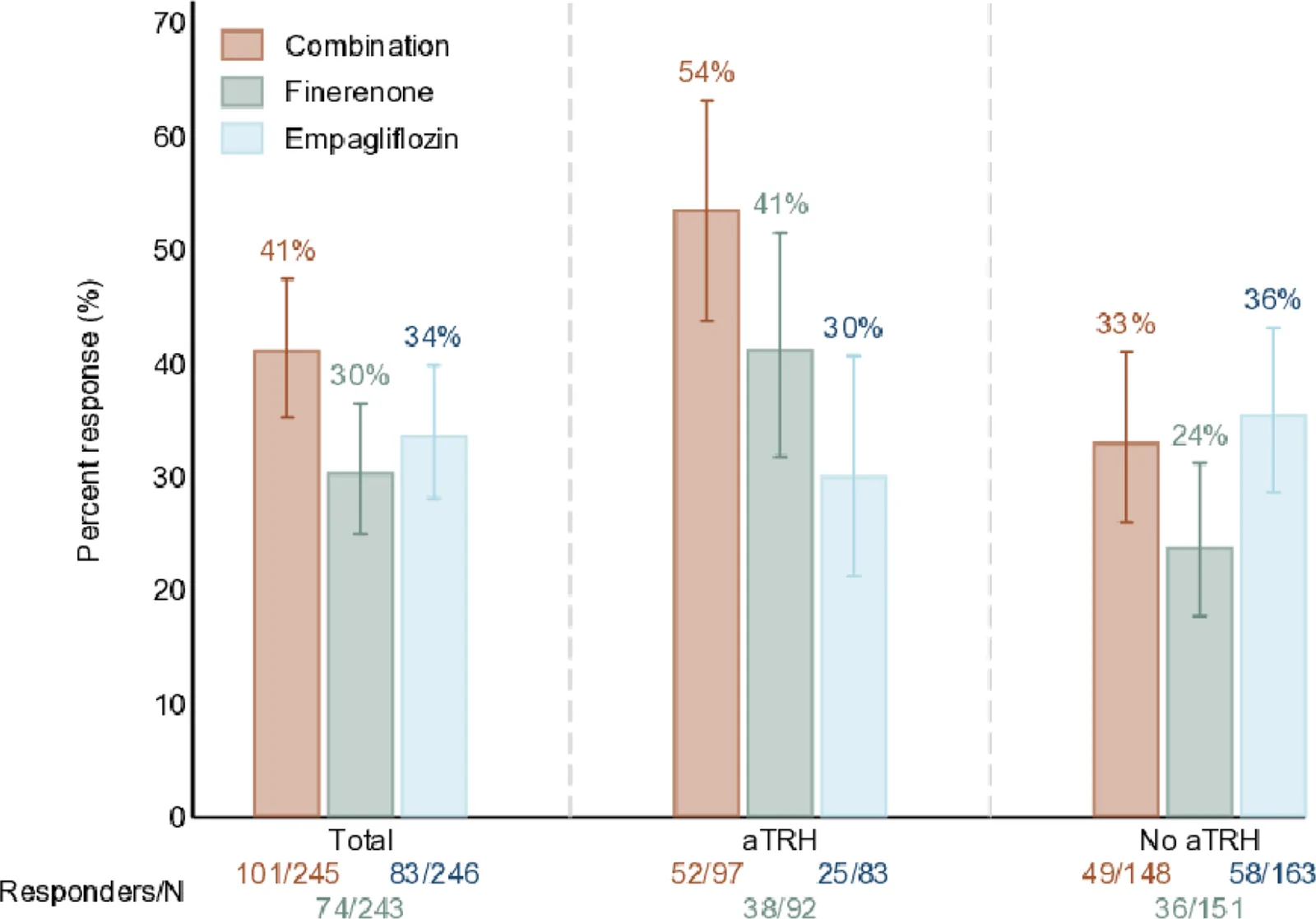
Percentage of Participants With Systolic Blood Pressure Reduction of at Least 10 mm Hg From Baseline to Day 180 (The Last Day of Treatment) Numbers at the bottom reflect the number of participants with response compared with the total number of participants in the group. Error bars are Wilson score 95% CIs for the percentage. aTRH = apparent treatment-resistant hypertension.

**Figure 2:**
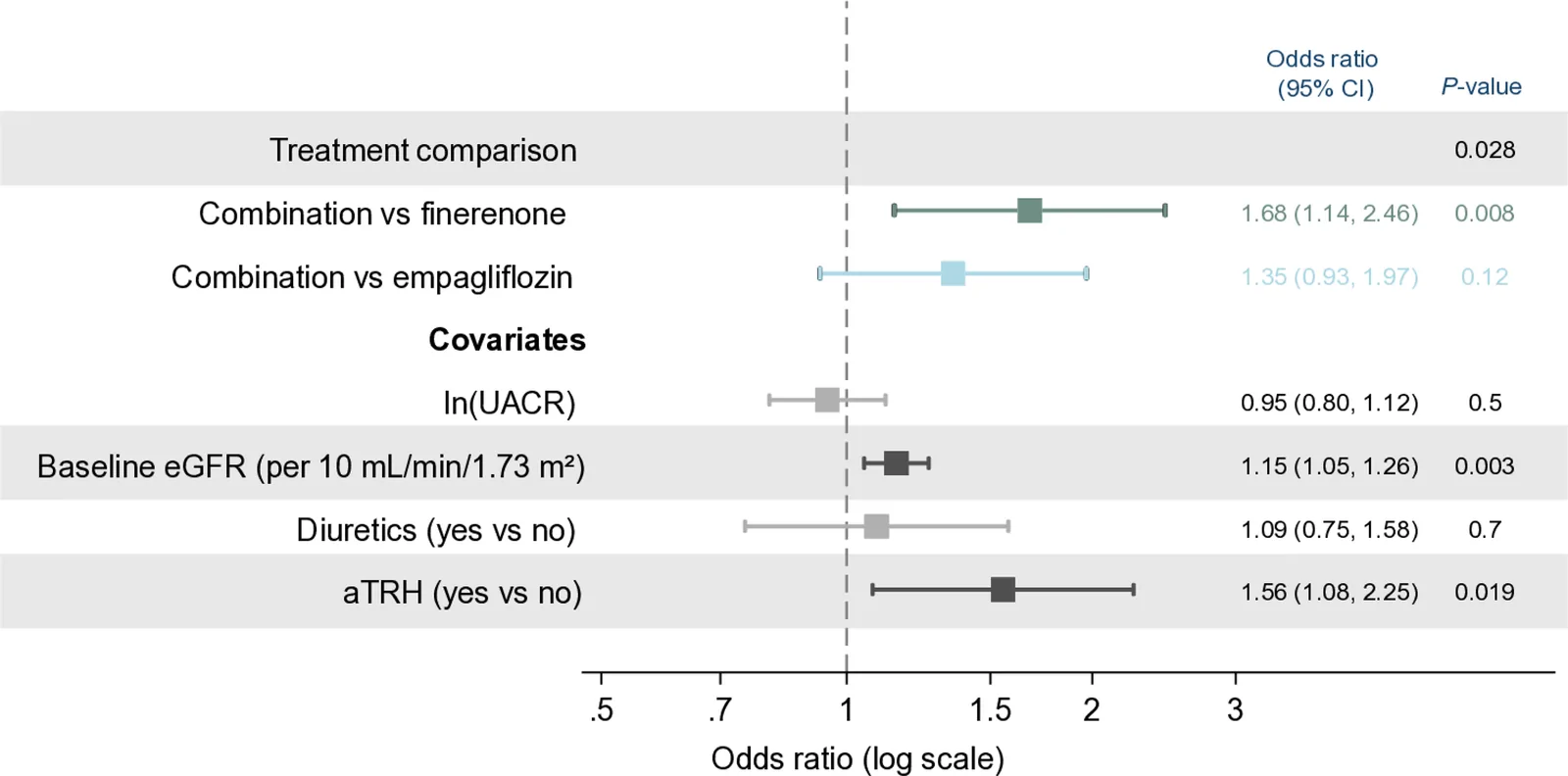
Clinical Determinants of a ≥10 mm Hg Drop in Systolic Blood Pressure From Baseline to Day 180 The squares represent the OR, and the horizontal bars the 95% CIs. Treatment, baseline eGFR, and aTRH were significant predictors (see text for details). eGFR = estimated glomerular filtration rate; ln = natural log; UACR = urinary albumin-to-creatinine ratio; other abbreviation as in Figure 1.

In the aTRH subgroup receiving combination therapy, least squares mean reductions in systolic BP were 8.7 mm Hg at day 14, 11.2 mm Hg at day 30, 8.8 mm Hg at day 90, and 9.3 mm Hg at day 180 (Supplemental Table 1). Figure 3 shows the marginal least squares mean change in systolic BP from baseline for each treatment group as a function of specific parameters (UACR, eGFR, diuretic use, and aTRH status), derived from the linear mixed models described previously. Although UACR (*P* = 0.98) and diuretic use (*P* = 0.17) were not associated with systolic BP reduction, both baseline eGFR (*P* = 0.005) and aTRH (*P* < 0.0001) were significantly associated with the change from baseline in systolic BP regardless of treatment. In other words, BP lowering with combination therapy increased with increasing baseline eGFR, and was greater in participants with aTRH compared with no aTRH. However, the aTRH effect was independent of treatment (treatment × aTRH interaction, *P* = 0.21).

**Figure 3:**
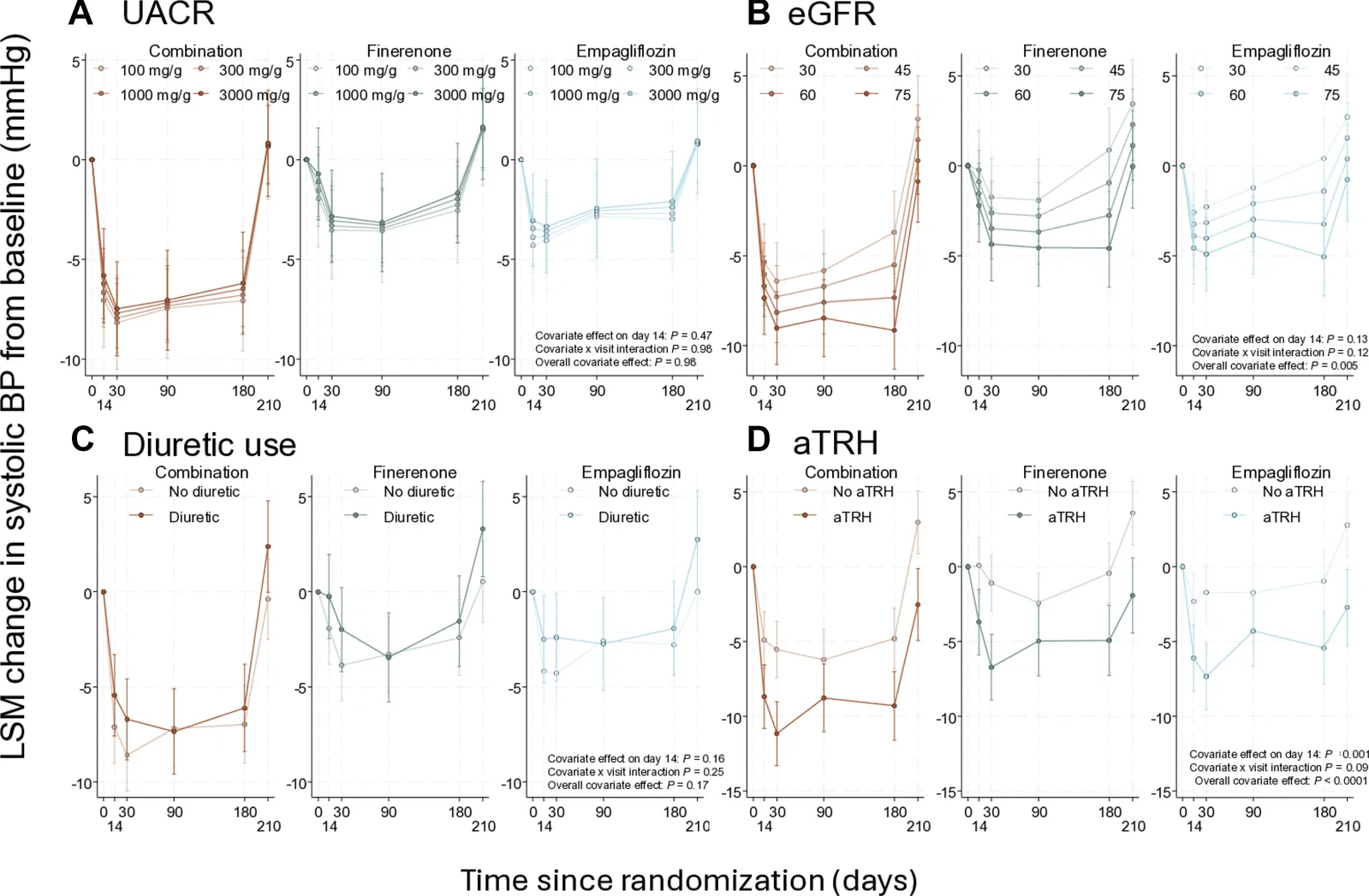
Least Squares Mean Change in Systolic Blood Pressure From Baseline to Day 210 by Baseline (A) UACR; (B) eGFR; (C) diuretic use; (D) aTRH. Figure panels display marginal least squares mean changes in systolic BP to day 210, adjusted for covariates at their means. Error bars are 95% CIs of the least squares mean. BP = blood pressure; LSM = least squares mean; other abbreviations as in Figures 1 and 2.

**Central Illustration:**
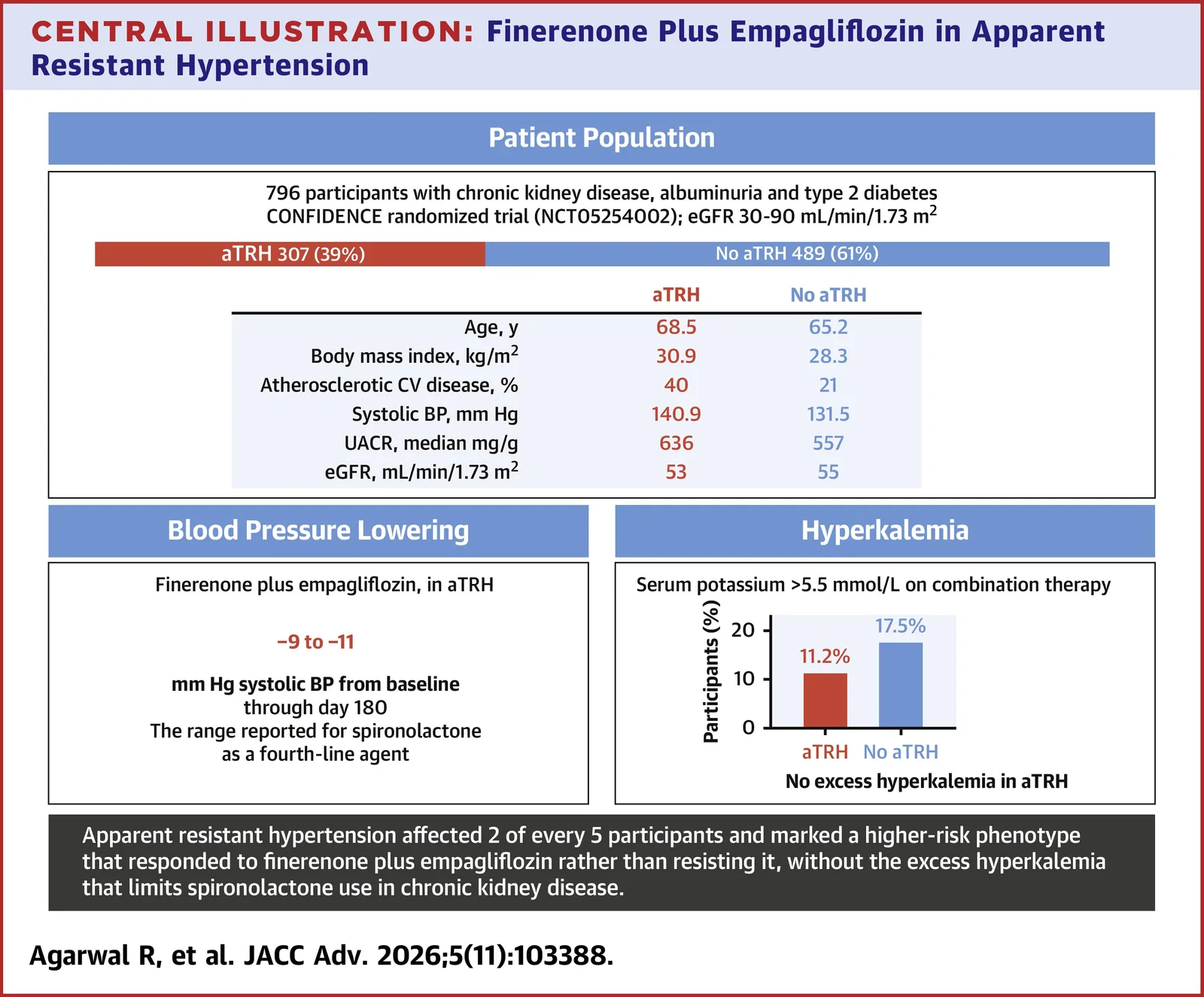
Finerenone Plus Empagliflozin in Apparent Resistant Hypertension Among 796 participants with chronic kidney disease, albuminuria, and type 2 diabetes randomized in the CONFIDENCE trial, 307 (39%) met criteria for apparent treatment-resistant hypertension (aTRH). Compared with participants without aTRH, they were older and had higher body mass index, more atherosclerotic cardiovascular disease, higher albuminuria, and higher systolic blood pressure (BP), with similar estimated glomerular filtration rate. Finerenone plus empagliflozin lowered systolic BP by 9 to 11 mm Hg from baseline through day 180 in the aTRH subgroup, the range reported for spironolactone as a fourth-line agent. Among participants receiving combination therapy, hyperkalemia (serum potassium >5.5 mmol/L) was less frequent in those with aTRH (11.2%) than in those without (17.5%). CV = cardiovascular; eGFR = estimated glomerular filtration rate; UACR = urinary albumin-to-creatinine ratio.

In sensitivity analyses, the addition of age, sex, race/ethnicity, BMI, and prior atherosclerotic cardiovascular disease as covariates to the mixed model did not materially alter the results: treatment effect *P* < 0.0001, aTRH *P* < 0.0001, eGFR *P* = 0.008, UACR *P* = 0.99, diuretic use *P* = 0.23, and treatment × aTRH interaction *P* = 0.19. Similarly, when the conventional definition of resistant hypertension requiring a diuretic among the ≥3 medications was applied, all findings were preserved: treatment *P* < 0.0001, resistant hypertension *P* = 0.011, eGFR *P* = 0.010, UACR *P* = 0.99, and treatment × resistant hypertension interaction *P* = 0.26.

When age, sex, race, BMI, and prior atherosclerotic cardiovascular disease were added to the logistic model (Supplemental Table 3), none predicted BP response except race, with higher odds of response in Black participants (OR: 1.97; 95% CI: 1.07-3.63; *P* = 0.029). The aTRH association was attenuated in that model (OR: 1.40; 95% CI: 0.95-2.05; *P* = 0.086); approximately half of the attenuation reflected the exclusion of 23 participants whose sparsely represented race categories perfectly predicted nonresponse, because the base model refitted on the same 696 participants gave an OR of 1.48 (*P* = 0.039). Substituting geographic region for race retained 705 participants and left aTRH independently associated with response (OR: 1.47; 95% CI: 1.01-2.16; *P* = 0.045). In the mixed model, race was likewise associated with the change in systolic BP (*P* < 0.0001), whereas age (*P* = 0.24), sex (*P* = 0.70), BMI (*P* = 0.11), and prior atherosclerotic cardiovascular disease (*P* = 0.089) were not.

### Influence of aTRH status on safety

Table 2 shows that aTRH was associated with an increased frequency of adverse events, serious adverse events, treatment-related drug events, and discontinuation of treatment due to adverse events; however, hyperkalemia was not more frequent in participants with aTRH compared with those without aTRH. Events of special interest, including acute kidney injury, genital mycotic infection, hyperkalemia, symptomatic hypotension, and urosepsis were similar in participants with aTRH (Table 3). Furthermore, the frequency of adverse events was generally not influenced disproportionately by treatment group (Supplemental Table 2).

**Table 2:** Summary of AEs by aTRH Subgroup

	No aTRH	aTRH	*P* Value
Any AE	250 (51.1)	196 (63.8)	<0.001
AE of special interest (TEAE)	2 (0.4)	5 (1.6)	0.115
Resulting in death (TEAE)	0 (0.0)	3 (1.0)	0.057
Resulting in death (all)	0 (0.0)	4 (1.3)	0.022
Any treatment-related SAE (TEAE)	25 (5.1)	27 (8.8)	0.041
Drug-related SAE (TEAE)	1 (0.2)	1 (0.3)	1.000
First occurrence of TEAE within participant	231 (47.2)	183 (59.6)	<0.001
Procedure-related AE (TEAE)	9 (1.8)	8 (2.6)	0.467
Related to drug treatment (TEAE)	55 (11.2)	57 (18.6)	0.004
Discontinued due to AE (TEAE)	15 (3.1)	15 (4.9)	0.190
SAE and discontinued due to AE (TEAE)	4 (0.8)	4 (1.3)	0.493
Hyperkalemia (TEAE)	39 (8.0)	26 (8.5)	0.804

Values are n (%). An AE that started or worsened after the first dose of study intervention up to 3 d after any temporary or permanent interruption of study intervention was considered a TEAE.

*P* values compare participants with and without aTRH by chi-square test, or Fisher exact test where fewer than 5 events occurred in either group.

AE = adverse event; SAE = serious adverse event; TEAE = treatment-emergent adverse event; other abbreviation as in Table 1.

**Table 3:** Summary of AEs of Special Interest by aTRH Subgroup

AE of Special Interest	Combo No aTRH (n = 160)	Combo aTRH (n = 107)	Fin No aTRH (n = 160)	Fin aTRH (n = 103)	Empa No aTRH (n = 169)	Empa aTRH (n = 97)	Total No aTRH (n = 489)	Total aTRH (n = 307)
Acute kidney injury	0 (0.0)	2 (1.9)	0 (0.0)	2 (1.9)	0 (0.0)	0 (0.0)	0 (0.0)	4 (1.3)
Genital mycotic infection	1 (0.6)	3 (2.8)	0 (0.0)	0 (0.0)	2 (1.2)	2 (2.1)	3 (0.6)	5 (1.6)
Hyperkalemia	28 (17.5)	12 (11.2)	28 (17.5)	20 (19.4)	13 (7.7)	12 (12.4)	69 (14.1)	44 (14.3)
Moderate hyperkalemia	23 (14.4)	11 (10.3)	24 (15.0)	19 (18.4)	10 (5.9)	11 (11.3)	57 (11.7)	41 (13.4)
Severe hyperkalemia	10 (6.3)	2 (1.9)	9 (5.6)	3 (2.9)	4 (2.4)	3 (3.1)	23 (4.7)	8 (2.6)
Symptomatic hypotension	2 (1.3)	1 (0.9)	0 (0.0)	0 (0.0)	0 (0.0)	0 (0.0)	2 (0.4)	1 (0.3)
Urosepsis	0 (0.0)	1 (0.9)	0 (0.0)	0 (0.0)	0 (0.0)	1 (1.0)	0 (0.0)	2 (0.7)
eGFR decline ≥30% day 30	11 (6.9)	6 (5.6)	4 (2.5)	6 (5.8)	0 (0.0)	3 (3.1)	15 (3.1)	15 (4.9)
eGFR decline ≥30% any time	32 (20.0)	26 (24.3)	26 (16.3)	19 (18.4)	14 (8.3)	15 (15.5)	72 (14.7)	60 (19.5)

Values are n (%). Hyperkalemia severity is based on central-laboratory serum potassium concentration: moderate, >5.5 to 6.0 mmol/L; severe, >6.0 mmol/L.

Combo = combination; Empa = empagliflozin; Fin = finerenone; other abbreviations as in Tables 1 and 2.

## Discussion

In this post hoc analysis of the CONFIDENCE trial among 796 participants with CKD (Central Illustration), albuminuria, and T2D, we report 3 principal findings. First, approximately 40% of participants met the functional definition of aTRH, and this subgroup exhibited a distinctly high-risk cardiometabolic phenotype. Second, combination therapy with finerenone and empagliflozin produced clinically meaningful systolic BP reductions in the aTRH subgroup, in the range of 9 to 11 mm Hg, comparable to the effect of spironolactone reported in PATHWAY-2,8 and this response persisted across sensitivity analyses, including use of a conventional definition of resistant hypertension requiring a diuretic, although the association was attenuated when race was included among the adjustment covariates (Supplemental Table 3). Third, although aTRH was associated with a higher burden of adverse events overall, hyperkalemia rates with combination therapy were paradoxically lower in the aTRH group (11.2%) than in the non-aTRH group (17.5%), supporting the safety of this combination in a population for whom spironolactone use is substantially limited by hyperkalemia risk.

The 40% prevalence of aTRH observed in the CONFIDENCE trial aligns with prior epidemiological data from CKD populations.^14^, ^15^ In the Chronic Renal Insufficiency Cohort study, the prevalence of aTRH escalated with declining kidney function, exceeding 54% in participants with stage 4 CKD.^17^ The clinical phenotype we describe (older age, higher BMI, greater atherosclerotic cardiovascular disease burden, higher UACR, and markedly increased antihypertensive polypharmacy) mirrors the well-characterized high-risk phenotype of aTRH in CKD.^1^, ^37^, ^38^ Collectively, these baseline differences confirm that aTRH within the CONFIDENCE cohort represents a higher-risk phenotype.

The pathophysiology of aTRH in CKD is primarily driven by impaired kidney sodium excretion, intravascular volume expansion triggered by MR overactivation, and inflammation.^18^, ^39^, ^40^ This mechanistic underpinning provides a strong rationale for why the combination of finerenone, a nonsteroidal MRA, and empagliflozin, which promotes natriuresis and osmotic diuresis,^28^, ^41^ should be particularly effective in this subgroup. We acknowledge that the higher baseline BP in the aTRH group was expected to be associated with larger absolute BP reductions, consistent with the Wilder principle and with our prior analysis demonstrating that baseline systolic BP was the strongest predictor of BP response magnitude in CONFIDENCE.^35^ The clinical relevance of this analysis, therefore, lies not in the observation that aTRH predicts larger BP reductions (which is mathematically expected) but in demonstrating that finerenone-empagliflozin combination therapy can safely deliver clinically meaningful BP lowering in this treatment-resistant population, positioning it as a tolerable alternative to spironolactone in CKD. Consistent with the mechanistic hypothesis, logistic regression demonstrated that the presence of aTRH independently predicted a ≥10 mm Hg reduction in systolic BP (OR: 1.56; 95% CI: 1.08-2.25; *P* = 0.019), even after adjustment for treatment group, baseline eGFR, UACR, and diuretic use. This finding suggests that the volume-dependent and MR-mediated mechanisms that underlie treatment resistance are the mechanisms targeted by this combination. Notably, although overall BP reduction was greater with combination therapy compared with either monotherapy, the relative advantage of combination over monotherapy did not differ by aTRH status (treatment × aTRH interaction, *P* = 0.21).

The BP reductions observed with combination therapy in the aTRH subgroup may be placed in broad clinical context alongside spironolactone, the established benchmark for resistant hypertension. The PATHWAY-2 trial demonstrated that spironolactone as add-on therapy reduced home systolic BP by approximately 8.7 mm Hg more than placebo in participants with resistant hypertension who had largely preserved kidney function (baseline mean eGFR 91.1 mL/min/1.73 m^2^).^8^ The overall reduction in systolic BP associated with combination therapy in CONFIDENCE participants with aTRH, which reached approximately 9 to 11 mm Hg from baseline, is in the same general range. However, we emphasize that direct quantitative comparison across these 2 trials is not valid given the important differences in study design (crossover vs parallel-group), BP measurement methodology (home vs office BP), and study populations (baseline mean eGFR 91 vs 53 mL/min/1.73 m^2^).^8^, ^9^, ^24^ However, our findings indicate that MRA-based combination therapy can achieve clinically meaningful BP reductions in CKD patients with aTRH—a population for whom spironolactone use is substantially limited by hyperkalemia, as the AMBER trial demonstrated.^9^, ^42^ By contrast, finerenone carries a substantially lower risk of hyperkalemia than spironolactone even without a potassium binder,^24^ and the present findings extend this advantage to the aTRH subgroup specifically.

A notable mechanistic insight from the regression analysis is that baseline eGFR, but not diuretic use or UACR, was a significant independent predictor of BP response (OR: 1.15 per 10 mL/min/1.73 m^2^; 95% CI: 1.05-1.26; *P* = 0.003). Although reduction in systolic BP increased with increasing eGFR, a BP response was still observed at lower eGFR (30 mL/min/1.73 m^2^) (Figure 3). This finding is consistent with evidence that sodium glucose co-transporter 2 inhibitors preserve their BP-lowering mechanisms even at lower GFRs through pathways independent of natriuresis, including sympathetic nervous system suppression and reduced arterial stiffness.^28^, ^29^ Conversely, the absence of a significant diuretic effect suggests that the BP benefit of this combination is independent of baseline diuretic use.

Adverse events and serious adverse events were more frequent in the aTRH group overall (63.8% vs 51.1% for any adverse event), this reflects the intrinsically higher comorbidity burden of this phenotype. Adverse events followed the usual pattern previously reported with finerenone and empagliflozin;^43^, ^44^ therefore, in the context of aTRH, the safety profile of finerenone plus empagliflozin or either drug alone did not demonstrate a treatment-specific harm signal. Hyperkalemia frequencies reported by the investigators were virtually identical between the aTRH and non-aTRH groups (8.5% vs 8.0%), and the pattern of adverse events across treatment arms was not disproportionately influenced by aTRH status. In the combination group hyperkalemia (serum potassium >5.5 mmol/L) frequencies were lower in the aTRH group (11.2%) compared with the no aTRH group (17.5%). This finding is clinically important. A common concern in prescribing MRA-based therapy in people already on multiple antihypertensives is that the added hemodynamic and electrolyte stress will amplify harm. The data indicate that this concern is not borne out in practice with the finerenone-empagliflozin combination.^32^, ^45^

Our findings should be interpreted in the context of an evolving therapeutic landscape for resistant hypertension. The recent Bax24 phase 3 trial demonstrated that baxdrostat, a selective aldosterone synthase inhibitor, produced a placebo-corrected reduction of 14.0 mm Hg in 24-hour ambulatory systolic BP in participants with confirmed resistant hypertension.^46^ Unlike MRAs, which block the MR downstream,^8^ aldosterone synthase inhibitors directly suppress aldosterone synthesis, thereby targeting the hormonal root of treatment resistance.^38^ These results reinforce the central role of aldosterone excess in the pathophysiology of resistant hypertension. Although the Bax24 trial excluded people with eGFR <45 mL/min/1.73 m^2^, baxdrostat increased the risk of hyperkalemia 4-fold compared with placebo. On the other hand, chlorthalidone in people with stage 4 CKD and resistant hypertension confirmed by 24-hour ambulatory BP monitoring produced a placebo-corrected, 13.9 mm Hg systolic BP reduction at 12 weeks^47^, ^48^; however, this came at the expense of an increased frequency of hypokalemia. In this context, the finerenone-empagliflozin combination in CKD might provide an alternative to the high risk of hyperkalemia or hypokalemia observed with emerging aldosterone synthase inhibitors and chlorthalidone, respectively.

### Study limitations

Several limitations of this analysis should be acknowledged. First, this is a post hoc, hypothesis-generating analysis; the CONFIDENCE trial was not designed or powered to test the role of aTRH as a modifier of treatment response. Second, participants were not prospectively enrolled based on aTRH status, and the analysis relies on a functional definition of aTRH using office-based BP and medication counts. Because ambulatory BP monitoring was not performed, we cannot distinguish true resistant hypertension from aTRH attributable to the white-coat effect,^3^ a distinction that proved critical in the Bax24 trial,^46^ where rigorous ambulatory confirmation excluded pseudoresistance. Third, medication adherence was not objectively assessed; nonadherence is a well-recognized contributor to aTRH and may have inflated the prevalence estimate.^5^, ^6^ Fourth, participants with BP ≥160/100 mm Hg were excluded by the trial protocol; consequently, our results apply to mild-to-moderate aTRH and should not be extrapolated to severe resistant hypertension without further study. These limitations underscore the need for prospective trials that specifically enroll participants with confirmed resistant hypertension to validate these findings. Fifth, in models further adjusted for demographic characteristics the association between aTRH and BP response was attenuated and no longer statistically significant when race was included, partly because sparsely represented race categories excluded 23 participants; these models were not prespecified and are exploratory.

## Conclusions

In participants with CKD, albuminuria, and T2D enrolled in the CONFIDENCE trial, approximately 40% met the criteria for aTRH. This subgroup exhibited a high-risk phenotype with a higher prevalence of cardiovascular comorbidities and greater albuminuria compared with participants without aTRH. The combination of finerenone and empagliflozin produced clinically meaningful BP reductions in the aTRH subgroup comparable in magnitude to those reported with spironolactone in PATHWAY-2, without the excess hyperkalemia that limits spironolactone use in CKD. This finding persisted across sensitivity analyses, including use of the conventional definition of resistant hypertension requiring a diuretic, although adjustment that included race attenuated the association (Supplemental Table 3). Importantly, unlike therapies evaluated solely for their BP-lowering effect, both finerenone and empagliflozin have established cardiovascular and kidney benefits in this population, and the CONFIDENCE trial demonstrated that their combination reduced albuminuria by approximately 30% more than either agent alone. Taken together, these results support the finerenone-empagliflozin combination as a clinically meaningful and tolerable strategy for managing resistant hypertension in people with CKD, particularly in patients for whom spironolactone is limited by hyperkalemia risk.^38^, ^46^, ^49^

Perspectives **COMPETENCY IN MEDICAL KNOWLEDGE:** Among participants with CKD, albuminuria, and type 2 diabetes in the CONFIDENCE trial, about 40% met the functional definition of apparent treatment-resistant hypertension (aTRH). These participants had a higher-risk cardiometabolic phenotype. In this group, finerenone-empagliflozin combination therapy lowered office systolic BP by about 9 mmHg at day 180, compared with about 5 mmHg in participants without aTRH. Hyperkalemia was not more frequent with aTRH. The greater response in aTRH is consistent with higher baseline BP.COMPETENCY IN PATIENT CARE AND PROCEDURAL SKILLS: In patients with CKD, type 2 diabetes, and aTRH, spironolactone is often limited by hyperkalemia. For these patients, combining finerenone and empagliflozin, which are already indicated for cardiorenal protection, provides added office BP lowering without excess hyperkalemia. Clinicians should still confirm true resistance with out-of-office BP measurement and adherence assessment.

**TRANSLATIONAL OUTLOOK:** Prospective trials should enroll patients whose resistant hypertension is confirmed by ambulatory BP monitoring and objective adherence testing, including patients with BP ≥160/100 mmHg. These trials should compare finerenone-empagliflozin with spironolactone, chlorthalidone, and aldosterone synthase inhibitors, using BP control and cardiorenal outcomes as endpoints.

## Funding support and author disclosures

The CONFIDENCE trial was funded by Bayer AG. CONFIDENCE was designed by the academic members of the executive committee in collaboration with representatives from Bayer. The sponsor, Bayer, was responsible for study supervision, site monitoring, and data collection. Primary data analysis was performed by Bayer and the senior author had separate data access and independently validated the analyses. All authors participated in the decision to submit the manuscript for publication with support from the study sponsor. Dr Agarwal has received personal fees and nonfinancial support from Bayer Healthcare Pharmaceuticals; personal fees and non-financial support from Akebia Therapeutics and Boehringer Ingelheim; serves as a member of data safety monitoring committees for Chinook, Renibus, Vera, and Vertex Pharmaceuticals; chairs the renal adjudication committees for trials conducted by Intercept and Alnylam pharmaceuticals; serves as Associate Editor for *Nephrology Dialysis Transplantation* and as an author for UpToDate; and receives research grants from the US Veterans Administration. Dr Navaneethan has received consulting fees from Alnylam, AstraZeneca, Bayer, Boehringer Ingelheim, Intercept, Novartis, and Vertex. Dr Correa-Rotter has received honoraria as consultant from AstraZeneca, Boehringer Ingelheim, Bayer, Chinook, AbbVie, Novo Nordisk, and Eli Lilly; research support from AstraZeneca, Boehringer Ingelheim, Roche, Eli Lilly, and Novo Nordisk; and has received honoraria/speaking fees from AstraZeneca, Boehringer Ingelheim, Novo Nordisk, Bayer, Silanes, and Amgen. Dr Estrella has received honoraria from Boehringer Ingelheim, AstraZeneca, and Bayer; and has a collaborative research agreement with Bayer on topics unrelated to the content of this paper. Dr Heerspink has received consulting fees from Alexion, AstraZeneca, Bayer, Boehringer Ingelheim, BioCity, Dimerix, Eli Lilly, Janssen, Novartis, Novo Nordisk, Roche, and Travere Therapeutics, all paid to his employer; research support from AstraZeneca, Boehringer Ingelheim, Bayer, Janssen, and Novo Nordisk, all paid to his employer; honoraria from AstraZeneca and Novo Nordisk; and travel expenses from AstraZeneca and Eli Lilly. Dr McGill is the principal investigator on grants to Washington University from Diamyd, Eli Lilly, Viking, Breakthrough T1D (formerly Juvenile Diabetes Research Foundation), and Novo Nordisk; has received royalties or licenses from Washington University; and has received consulting fees from Bayer, Mannkind, Novo Nordisk, Pfizer, and Vertex. Dr Mottl has received research support and personal fees from Alexion, Boehringer Ingelheim, Bayer, Chinook Therapeutics, and Novartis, paid to her institution; royalties from UpToDate; consulting fees from Bayer, Otsuka, Vera, and Chinook/Novartis; and travel support and payment for educational symposium from Novo Nordisk. Dr Nangaku has received research grants from Astellas, Bayer, Boehringer Ingelheim, Chugai, Daiichi Sankyo, JT, Kyowa-Kirin, Takeda, Tanabe-Mitsubishi, and Torii; consulting fees from Astellas, Boehringer Ingelheim, Daiichi Sankyo, JT, Kyowa-Kirin, and Tanabe-Mitsubishi; and honoraria from Astellas, AstraZeneca, Boehringer Ingelheim, Chugai, Daiichi Sankyo, GSK, Kyowa-Kirin, and Tanabe-Mitsubishi. Dr Rosenstock has received research grants from Amgen, Applied Therapeutics, AstraZeneca, Biomea Fusion, Boehringer Ingelheim, Carmot, Corcept, Eli Lilly, Hanmi, Merck, Novartis, Novo Nordisk, Oramed, Regeneron, Pfizer, and Sanofi; served on scientific advisory boards and received honorarium or consulting fees from Amgen, Applied Therapeutics, Biomea Fusion, Eccogene, Eli Lilly, Hanmi, Novo Nordisk, Regeneron, Regor, Roche, Sanofi, and Structure Therapeutics; and received honoraria for lectures from Eli Lilly, Novo Nordisk, and Sanofi. Dr Rossing has received research support from AstraZeneca, Bayer, and Novo Nordisk, all made out to his institution; consulting fees from Abbott, Amgen, Astellas, Bayer, Boehringer Ingelheim, Eli Lilly, Roche, and Gilead, all made out to his institution; payment or honoraria from Daiichi Sankyo, made out to his institution; support for attending meetings and/or travel from Bayer; and receipt of study drug for an investigator-initiated study from Bayer, AstraZeneca, Novo Nordisk, and Lexicon Pharma. Dr Vaduganathan has received research grant support, served on advisory boards, or had speaker engagements with Alnylam Pharmaceuticals, American Regent, Amgen, AstraZeneca, Bayer AG, Baxter Healthcare, Bristol Myers Squibb, Boehringer Ingelheim, Chiesi, Cytokinetics, Esperion, Fresenius Medical Care, Idorsia Pharmaceuticals, Lexicon Pharmaceuticals, Merck, Milestone Pharmaceuticals, Novartis, Novo Nordisk, Pharmacosmos, Relypsa, Roche Diagnostics, Sanofi, and Tricog Health; and participates on clinical trial committees for studies sponsored by Amgen, AstraZeneca, Boehringer Ingelheim, Galmed, Novartis, Bayer AG, Occlutech, Pharmacosmos, and Impulse Dynamics. Mr Scott is an employee of Bayer Healthcare Inc, USA. Dr Li is an employee of Bayer AG, Germany. Dr Brinker is an employee of Bayer AG, Germany, and is named on a pending patent for finerenone for the treatment of CKD in type 1 diabetes. Dr Morales has been a speaker for Amgen, Bayer, Boehringer Ingelheim, Eli Lilly, Novo Nordisk, and Phathom Pharmaceuticals; and a consultant and advisory board member for Bayer, Boehringer Ingelheim and Eli Lilly. Dr Handelsman has received research grants, and consultant and speaker honoraria from Amgen, AstraZeneca, Bayer, Boehringer Ingelheim, Corcept, Ionis, Merck, Novo Nordisk, Regeneron, Sanofi, and Verdiva Bio. Dr Green has received funds for research, paid to her institution, from Bluedrop, Boehringer Ingelheim, Eli Lilly, Merck, Roche, Cour, and GentiBio; is a consultant for AstraZeneca, Bayer, Boehringer Ingelheim, Corcept, Eli Lilly, Idorsia, Mineralys, Novo Nordisk, Sparrow, Valo, Vertex, and Zealand; and holds a leadership or fiduciary role with the American Diabetes Association. Dr Mann has received grants from McMaster University (Hamilton, Canada) and Novo Nordisk; consulting fees from Bayer and Novo Nordisk; honoraria from AstraZeneca, Bayer, Novartis, UpToDate, and Novo Nordisk; has participated on a data safety monitoring board or advisory board for Cytel, Icon, IQVIA, Parexel, WCG, and Sanofi; and had a leadership role in the Kidney Disease: Improving Global Outcomes group. Dr Weir has served as a scientific advisor for AstraZeneca, Bayer, CSL Vifor, Corcept, Mineralys, Novo Nordisk, Idorsia, and Vera.
