# Supplementary material for: Finerenone-Empagliflozin Combination Therapy in Apparent Resistant Hypertension: A CONFIDENCE Trial Post Hoc Analysis
Source: JACC Adv. 2026 Oct 23;5(11):103388. doi: 10.1016/j.jacadv.2026.103388 (PMC13721014; doi:10.1016/j.jacadv.2026.103388)
Supplement: Supplementary file 1 [file mmc1.docx]

# Supplemental Material

**Finerenone-Empagliflozin Combination Therapy in Apparent Resistant Hypertension**

**A CONFIDENCE Trial Post Hoc Analysis**

Rajiv Agarwal, MD, MS; Sankar D. Navaneethan, MD, MS, MPH; Ricardo Correa-Rotter, MD; Michelle M. Estrella, MD, MHS; Hiddo J. L. Heerspink, PhD; Janet B. McGill, MD; Amy K. Mottl, MD; Masaomi Nangaku, MD, PhD; Julio Rosenstock, MD; Peter Rossing, MD; Muthiah Vaduganathan, MD, MPH; Javier Morales, MD; Yehuda Handelsman, MD; Jennifer B. Green, MD; Johannes F.E. Mann, MD; Charlie Scott, MSc; Li Li, MD, MBA; Meike Brinker, MD; Matthew R. Weir, MD

**Correspondence**:

Rajiv Agarwal, MD, MS
Richard L. Roudebush VA Medical Center
Indiana University School of Medicine
1481 W. 10th St, 111N, Indianapolis, IN 46202, USA
Tel: +1 317-988-2241; Email: [ragarwal@iu.edu](mailto:ragarwal@iu.edu)

[Supplemental Material	1](#_Toc231291806)

[TABLE S1 Least Squares Mean (95% CI) Change in Systolic BP From Baseline to Day 210 by Treatment Group and aTRH	2](#_Toc231291807)

[TABLE S2 AEs by Treatment Stratified by aTRH	3](#_Toc231291808)

## TABLE S1 Least Squares Mean (95% CI) Change in Systolic BP From Baseline to Day 210 by Treatment Group and aTRH Subgroup

| Treatment/visit | Overall | No aTRH | aTRH |
| --- | --- | --- | --- |
| Combination |  |  |  |
| Day 14 | −6.4 (−8.0 to −4.7) | −4.9 (−6.8 to −3.0) | −8.7 (−10.8 to −6.6) |
| Day 30 | −7.7 (−9.4 to −6.1) | −5.5 (−7.4 to −3.7) | −11.2 (−13.3 to −9.0) |
| Day 90 | −7.2 (−8.9 to −5.4) | −6.2 (−8.2 to −4.2) | −8.8 (−11.0 to −6.5) |
| Day 180 | −6.5 (−8.3 to −4.7) | −4.8 (−6.8 to −2.8) | −9.3 (−11.6 to −7.0) |
| Day 210 | 0.8 (−1.1 to 2.6) | 3.0 (0.9 to 5.1) | −2.5 (−4.9 to −0.1) |
| Finerenone |  |  |  |
| Day 14 | −1.3 (−3.0 to 0.3) | 0.1 (−1.8 to 2.0) | −3.7 (−5.9 to −1.5) |
| Day 30 | −3.2 (−4.9 to −1.5) | −1.1 (−3.0 to 0.8) | −6.7 (−8.9 to −4.5) |
| Day 90 | −3.4 (−5.1 to −1.6) | −2.4 (−4.4 to −0.4) | −5.0 (−7.3 to −2.6) |
| Day 180 | −2.1 (−3.9 to −0.3) | −0.4 (−2.4 to 1.6) | −4.9 (−7.3 to −2.6) |
| Day 210 | 1.5 (−0.4 to 3.4) | 3.6 (1.5 to 5.7) | −1.9 (−4.4 to 0.6) |
| Empagliflozin |  |  |  |
| Day 14 | −3.7 (−5.3 to −2.0) | −2.3 (−4.2 to −0.5) | −6.1 (−8.3 to −3.9) |
| Day 30 | −3.7 (−5.4 to −2.1) | −1.7 (−3.6 to 0.1) | −7.3 (−9.6 to −5.1) |
| Day 90 | −2.7 (−4.5 to −1.0) | −1.7 (−3.7 to 0.2) | −4.3 (−6.6 to −1.9) |
| Day 180 | −2.6 (−4.4 to −0.8) | −0.9 (−2.9 to 1.0) | −5.4 (−7.8 to −3.0) |
| Day 210 | 0.8 (−1.0 to 2.7) | 2.8 (0.7 to 4.9) | −2.7 (−5.2 to −0.2) |

Mixed model adjusted for baseline ln(UACR), eGFR, and diuretic use, estimated in 767 of the 796 participants who contributed at least 1 post-baseline measurement (3,646 observations).
aTRH = apparent treatment-resistant hypertension; BP = blood pressure; CI = confidence interval; eGFR = estimated glomerular filtration rate; UACR = urinary albumin-to-creatinine ratio.

## TABLE S2 AEs by Treatment Stratified by aTRH Subgroup

|  | Combination | | Finerenone | | Empagliflozin | | Total | |
| --- | --- | --- | --- | --- | --- | --- | --- | --- |
| Clinical outcome — n (%) | No aTRH | aTRH | No aTRH | aTRH | No aTRH | aTRH | No aTRH | aTRH |
| Any AE | 90 (56.3) | 61 (57.0) | 76 (47.5) | 73 (70.9) | 84 (49.7) | 62 (63.9) | 250 (51.1) | 196 (63.8) |
| AE of special Interest (TEAE) | 2 (1.3) | 3 (2.8) | 0 (0.0) | 2 (1.9) | 0 (0.0) | 0 (0.0) | 2 (0.4) | 5 (1.6) |
| Resulting in death (TEAE) | 0 (0.0) | 1 (0.9) | 0 (0.0) | 0 (0.0) | 0 (0.0) | 2 (2.1) | 0 (0.0) | 3 (1.0) |
| Resulting in death (all) | 0 (0.0) | 2 (1.9) | 0 (0.0) | 0 (0.0) | 0 (0.0) | 2 (2.1) | 0 (0.0) | 4 (1.3) |
| Any treatment-related SAE (TEAE) | 10 (6.3) | 9 (8.4) | 8 (5.0) | 8 (7.8) | 7 (4.1) | 10 (10.3) | 25 (5.1) | 27 (8.8) |
| Drug-related SAE (TEAE) | 1 (0.6) | 0 (0.0) | 0 (0.0) | 1 (1.0) | 0 (0.0) | 0 (0.0) | 1 (0.2) | 1 (0.3) |
| First occurrence of TEAE within participant | 85 (53.1) | 58 (54.2) | 69 (43.1) | 67 (65.0) | 77 (45.6) | 58 (59.8) | 231 (47.2) | 183 (59.6) |
| Procedure-related AE (TEAE) | 5 (3.1) | 4 (3.7) | 2 (1.3) | 2 (1.9) | 2 (1.2) | 2 (2.1) | 9 (1.8) | 8 (2.6) |
| Related to drug treatment (TEAE) | 24 (15.0) | 22 (20.6) | 17 (10.6) | 17 (16.5) | 14 (8.3) | 18 (18.6) | 55 (11.2) | 57 (18.6) |
| Discontinued due to AE (TEAE) | 8 (5.0) | 4 (3.7) | 4 (2.5) | 5 (4.9) | 3 (1.8) | 6 (6.2) | 15 (3.1) | 15 (4.9) |
| SAE and discontinued due to AE (TEAE) | 2 (1.3) | 1 (0.9) | 1 (0.6) | 2 (1.9) | 1 (0.6) | 1 (1.0) | 4 (0.8) | 4 (1.3) |
| Hyperkalemia (TEAE) | 18 (11.3) | 7 (6.5) | 17 (10.6) | 13 (12.6) | 4 (2.4) | 6 (6.2) | 39 (8.0) | 26 (8.5) |

An AE that started or worsened after the first dose of study intervention up to 3 days after any temporary or permanent interruption of study intervention was considered a TEAE.
AE = adverse event; aTRH = apparent treatment-resistant hypertension; SAE = serious adverse event; TEAE = treatment-emergent adverse event.

## TABLE S3 Predictors of a ≥10 mmHg Reduction in Systolic BP at Day 180

**Panel A. Odds ratios (95% CI) and P values**

|  | Model 1: base | | Model 2: + demographics (race) | | Model 3: + demographics (region) | |
| --- | --- | --- | --- | --- | --- | --- |
| Predictor | OR (95% CI) | P | OR (95% CI) | P | OR (95% CI) | P |
| Combination vs finerenone | 1.68 (1.14–2.46) | 0.008 | 1.65 (1.11–2.44) | 0.013 | 1.62 (1.10–2.39) | 0.015 |
| Combination vs empagliflozin | 1.35 (0.93–1.97) | 0.117 | 1.42 (0.97–2.09) | 0.073 | 1.39 (0.95–2.04) | 0.093 |
| ln(UACR), per 1 unit | 0.95 (0.80–1.12) | 0.518 | 0.99 (0.84–1.18) | 0.942 | 0.98 (0.82–1.16) | 0.773 |
| Baseline eGFR, per 10 mL/min/1.73 m² | 1.15 (1.05–1.26) | 0.003 | 1.14 (1.04–1.26) | 0.005 | 1.15 (1.05–1.27) | 0.003 |
| Diuretic use, yes vs no | 1.09 (0.75–1.58) | 0.656 | 1.03 (0.69–1.53) | 0.882 | 1.05 (0.71–1.56) | 0.795 |
| aTRH, yes vs no | 1.56 (1.08–2.25) | 0.019 | 1.40 (0.95–2.05) | 0.086 | 1.47 (1.01–2.16) | 0.045 |
| Age, per year | — |  | 1.01 (0.99–1.03) | 0.284 | 1.01 (0.99–1.02) | 0.511 |
| Male sex, vs female | — |  | 1.04 (0.72–1.52) | 0.828 | 0.98 (0.68–1.43) | 0.932 |
| Race: Black vs White | — |  | 1.97 (1.07–3.63) | 0.029 | — |  |
| Race: Asian vs White | — |  | 0.92 (0.61–1.38) | 0.675 | — |  |
| Region: Europe vs North America | — |  | — |  | 0.89 (0.58–1.38) | 0.612 |
| Region: Asia vs North America | — |  | — |  | 0.75 (0.48–1.17) | 0.202 |
| Body mass index, per kg/m² | — |  | 1.00 (0.97–1.04) | 0.831 | 1.00 (0.97–1.03) | 0.883 |
| Prior atherosclerotic CV disease, yes vs no | — |  | 1.23 (0.85–1.78) | 0.272 | 1.20 (0.83–1.73) | 0.329 |

**Panel B. The aTRH odds ratio across all five fits, separating covariate adjustment from the change in analyzed sample**

| Model | N | aTRH OR (95% CI) | P |
| --- | --- | --- | --- |
| Base model (as shown in Figure 2) | 719 | 1.56 (1.08–2.25) | 0.019 |
| Base model, refit on the Model 2 sample | 696 | 1.48 (1.02–2.15) | 0.039 |
| Model 2: + age, sex, race, BMI, prior ASCVD | 696 | 1.40 (0.95–2.05) | 0.086 |
| Base model, refit on the Model 3 sample | 705 | 1.53 (1.06–2.22) | 0.024 |
| Model 3: + age, sex, region, BMI, prior ASCVD | 705 | 1.47 (1.01–2.16) | 0.045 |

All models estimate the odds of a ≥10 mmHg reduction in systolic blood pressure from baseline to day 180. The base model is the model shown in Figure 2. Treatment odds ratios are expressed with combination therapy as the active comparator. Model 2 analyzes 696 of 719 participants because 4 participants in the American Indian and Native Hawaiian categories perfectly predicted non-response and were dropped, together with 19 with missing covariates; Model 3 substitutes geographic region for race and retains 705. Panel B refits the base model on each of those samples so that adjustment can be distinguished from the change in sample. In the corresponding linear mixed model of systolic blood pressure change, race was associated with the outcome (P < 0.0001), whereas age (P = 0.24), sex (P = 0.70), body mass index (P = 0.11) and prior atherosclerotic cardiovascular disease (P = 0.089) were not. These models were not pre-specified and are exploratory.
ASCVD = atherosclerotic cardiovascular disease; aTRH = apparent treatment-resistant hypertension; BMI = body mass index; CI = confidence interval; CV = cardiovascular; eGFR = estimated glomerular filtration rate; OR = odds ratio; UACR = urinary albumin-to-creatinine ratio.
